# Spiritual leadership among nursing educators: a correlational cross-sectional study with psychological capital

**DOI:** 10.1186/s12912-022-01163-y

**Published:** 2022-12-31

**Authors:** Mennat-Allah G. Abou Zeid, Ayman Mohamed El-Ashry, Manar Ahmed Kamal, Mahmoud Abdelwahab Khedr

**Affiliations:** 1 Lecturer, Nursing Administration Department, Faculty of Nursing, Ain Shams University, Cairo, Egypt; 2 Lecturer, Psychiatric Nursing and Mental Health Department, Faculty of Nursing, Alexandria University, Alexandria, Egypt; 3 Faculty of Medicine, Benha University, Benha, Egypt; 4 Lecturer, Psychiatric Nursing and Mental Health Department, Faculty of Nursing, Alexandria University, Alexandria, Egypt

**Keywords:** Spiritual leadership, Psychological capital, Nursing educators

## Abstract

**Background:**

Spiritual leadership is the most positive leadership style accepted by positive organizational behavior scholars that contributes to psychological capital. Spiritual leadership could be a critical organizational resource for followers. The study aims to examine the levels of spiritual leadership and psychological capital among nursing educators and determine the relationship between spiritual leadership and psychological capital among nurse educators.

**Methods:**

A cross-sectional design was applied using two tools: spiritual leadership and psychological capital scales. Over 3 months, data were collected from 213 nursing educators.

**Results:**

The spiritual leadership level is moderate to high (49.8 and 38.5%). The psychological capital level is high (63.4%). The association between spiritual leadership and psychological capital is a strong, positive, and highly significant correlation (R-value = 0.63, *P* = 0.001). The regression analysis predicted that male participants were expected to have more spiritual leadership and psychological capital than females.

**Conclusion:**

It can be concluded and validated how important it is to develop and practice spiritual leadership to foster followers’ psychological capital.

**Implications of nursing management:**

as nursing leaders must have spiritual competencies to promote workplace spirituality on the daily agenda as a foundational area in management.

**Supplementary Information:**

The online version contains supplementary material available at 10.1186/s12912-022-01163-y.

## Background

Nursing leaders’ roles have recently expanded to promote the well-being and satisfaction of nurses and patients. Recent incidents have reignited the need for cultural institutions to become more caring and empathetic [1]. Nurse educators are crucial in developing the workforce, serving as mentors, and providing the leadership necessary to integrate evidence-based practice and enhance patient outcomes [2]. Moreover, the vital characteristics of a good nurse educator’s personality are to be compassionate, dynamic, supportive, caring, empathetic, challenging, and knowledgeable [3].

Significant global, cultural, and organizational change has occurred due to the problematic workplace climate of the 20-first century [4]. A widespread confidence crisis right now affects both employees and institutions worldwide. In line with that, organizations have begun to emphasize employee optimism and building strong character traits more than employee negativity and deficiencies [5]. Academics and organizational behavior specialists have similarly begun to emphasize the positive aspects of organizational life. This shift in mentality and psychological capital necessitates a more holistic leadership style that can integrate the minds and souls of people: namely, spiritual leadership, which was addressed by the current study [6].

### Spiritual leadership

In history’s scholarly communities, spiritual leadership has no unified definition. However, scholars from various cultural backgrounds have begun to agree with Fry’s point of view: spiritual leaders can establish an organizational vision and organizational values based on individual and team connections and combine their attitudes, values, and behaviors to make employees feel the importance of their work, as well as appreciation and understanding of the organization [4].

Numerous studies show that work-based spiritual leadership efforts provide excellent results because spiritual leaders help their followers develop a strong calling by creating and communicating a clear and inspirational vision, strengthening their beliefs, and caring for others [7]. Furthermore, spiritual leaders feel compassion and value for employees, pay attention to upper and lower feedback during interactions with them, and do their best to respond to the demands of employees, resulting in a healthier working environment and a climate in which individuals may express themselves autonomously [8, 9**].**

### Psychological capital

Psychological capital is intellectual satisfaction that empowers the person’s behavior toward the ability to face problems during their work [10]. Numerous studies reveal positive outcomes with work-based psychological capital. Employees with a high level of psychological capital can perform better, whereas those with poor psychological capital are depressed, pressured, and anxious, and consequently, they are unable to function as required, and as a result, they are unsatisfied with their jobs [11].

In addition, Lupşa et al., (2020) suggested that psychological capital is open to change and is developable, which is why it is considered to be a “state-like” rather than a “trait-like” feature. It can be modified and improved with the help of positive organizational interventions, programs, and on-the-job training [12]. As Hameed, Bano, & Ahmed (2022) concluded, academics with psychological capital can face challenges, adopt changes, exhibit positive behavior in critical situations, and expect positive results in the future, which make them satisfied with their jobs [13].

### Theoretical background

From the perspective of the job demands-resources (JD-R) theory and spiritual leadership theory (SLT), it is very clear how psychological capital is influenced by spiritual leadership [4, 13]. According to the JD-R model and SLT, spiritual leadership can enhance followers’ psychological capital, resulting in a higher job engagement. A person’s positive psychological condition of development, marked by self-efficacy, optimism, hope, and resiliency, is referred to as psychological capital [14]. Spiritual leaders will likely create a welcoming and caring environment by treating their followers with concern, respect, and confidence [15]. Employee psychological capital can be developed in a supportive organizational context [16].

The SLT model essentially differentiates between two types of spiritual well-being: calling and membership. The source of incentive calling is found in the workplace, but the motivation for membership is the organization [15]. Employers view work as a job or profession when they are busy working every day. Some individuals may only view work as a means of making money or moving up the corporate ladder. However, people may also see employment as a way to fulfill their needs, discover their purpose in life, or give back to the community. Researchers categorize these individuals as having a sense of calling while working [4, 17]. “The experience of transcendence” or “how one makes a difference by serving others and, in doing so, finds meaning and purpose in life” is what calling is described as [4]. According to SLT, spiritual leaders can provide their followers with a sense of purpose, meaning, and the ability to make a difference, and develop a meaningful vision. Spiritual leaders can improve their followers’ sense of calling at work and their psychological capital [4, 18].

Furthermore, according to SLT, calling is an essential mechanism through which spiritual leaders promote employees’ positive outcomes. Calling shows that people’s perceptions of their well-being, such as their sense of purpose in life or their level of contentment, are positively correlated with psychological capital [19]. Also, self-efficacy is one of the crucial elements of psychological capital, and studies have shown that calling is positively associated with it [20]. Finally, calling is positively linked to job satisfaction. Employees are more likely to feel happy and optimistic when they are more satisfied at work. Positive emotions often lead to more significant psychological capital development in individuals [21, 22].

### Significance of the study

Universities play a vital role in human capital education. It points out that the efficiency and effectiveness of the country’s educational systems promote overall development and growth. Nurse educators are one of the most significant resources in any society, one of the most essential factors in educational systems, and they play a crucial role in training specialized forces. Therefore, achieving good psychological capital is very important; spiritual leadership can be improved by changing and manipulating psychological capital factors, thus moving toward the organization’s development [23]. Spiritual leadership plays a significant role in educational organizations that aim to raise successful nurse educators in every sphere of life. This is because academic life includes a mix of interactions between the spiritual and physical worlds**.** Nurse educators who view work as a spiritual expression will have a more positive outlook, contribute more, create better working conditions effectively, and lead to a better quality of work life [24].

Therefore, the two issues of spiritual leadership and psychological capital in the workplace are complementary. However, only a few studies have looked at this relationship worldwide. Therefore, knowledge of these variables is vital when designing interventions or developing organizational policies to enhance spiritual leadership behavior and psychological capital among nursing educators. Therefore, the present study was conducted to examine the levels of spiritual leadership and psychological capital among nursing educators and to determine the relationship between spiritual leadership and psychological capital among nurse educators. Moreover, this study is the first to examine the relationship between spiritual leadership and psychological capital among nursing educators in Egypt.

### Study design

A cross-sectional study was conducted in all academic departments at the Faculty of Nursing, Alexandria University, Egypt. It includes Medical and Surgical Nursing, Pediatric Nursing, Obstetrics and Gynecology Nursing, Psychiatric and Mental Health Nursing, Nursing Administration, Community Health Nursing, Critical Care and Emergency Nursing, Nursing Education, and Geriatric Nursing.

### Participants: sample size calculation and sampling technique

The researchers in the present study used convenient cluster sampling to select participants for recruitment from all academic positions (approximately 35 participants from each position). Academic positions at the faculty of nursing, Alexandria University, Egypt, include about 372 nurse educators, distributed as follows: professors (75), assistant professors (62), lecturers (88), assistant lecturers (60), demonstrators, and practical guides (87). For sample size calculation, the total population of nursing educators was 372, with a 5% acceptable error, a 50% expected frequency, and a 95% confidence coefficient. The Epi-Info Program indicated that the minimal sample size should be 190. The chosen number of recruited participants in the present study reached 213.

The inclusion criteria consisted of the subjects of the study who work in the previously mentioned departments with more than 6 months of experience, currently teaching or providing educational services for nursing students, full-time employment (at least 20 hours of work per week), were available during the time of data collection, and the subjects of the study showed an interest in participating in this study. Whereas all nursing educators who didn’t have the previous characteristics were excluded.

### Data collection

The researchers used a three-section-based questionnaire for data collection (Supplementary file).

Section 1. Socio-demographics: sex, age, marital status, number of children, residence, educational qualifications, academic position, department, years of experience since bachelor of nursing graduation, and years of experience in current position.

Section 2. Spiritual Leadership Questionnaire (SLQ): Fry et al., (2005) developed it to assess spiritual leadership levels among nurse educators. It consisted of 17 items grouped under three dimensions, like the following: Vision consisted of five items; Hope/Faith consisted of five items. Finally, altruistic love consisted of seven items. The scoring system of SLQ was a five-point Likert scale ranging from strongly agree=(5) to strongly disagree=(1) . Items were scored 5, 4, 3, 2, and 1 for the responses “strongly agree,” “agree,” “neutral,” “disagree,” and “strongly disagree,” respectively [18].

The overall score of the spiritual leadership scale and its dimensions were measured based on five points Likert Scale ranging and grading as (> 75%) indicated high agreements on the overall spiritual leadership scale and its dimensions, (50% - < 75%) indicated moderate agreements on overall spiritual leadership scale and its dimensions, (< 50%) indicated low agreements on overall spiritual leadership scale and its dimensions. The Cronbach’s alpha reliability estimate in the present study was 0.961, and that of the original study was 0.980 [18].

Section 3. Psychological Capital Questionnaire (PCQ): The 24-item Psychological Capital Questionnaire (PCQ) developed by Luthans, Youssef, and Avolio, (2017) was utilized in this study. The instrument consisted of four constructs: self-efficacy, hope, reliance, and optimism. Additionally, each construct was measured based on six items. The scoring system of SLQ was a five-point Likert scale ranging from strongly agree= (5)to strongly disagree=(1). Items were scored 5, 4, 3, 2, and 1 for the responses “strongly agree,” “agree,” “neutral,” “disagree,” and “strongly disagree,” respectively [25].

The overall scores were measured based on five points Likert Scale ranging and grading as (> 75%) indicated high agreements on the overall psychological capital scale and its dimensions, (50 - < 75%) indicated moderate agreements on the overall psychological capital scale and its dimensions, (< 50%) indicated low agreements on the overall psychological capital scale and its dimensions. The Cronbach’s alpha reliability estimate in the present study was 0.965, and in another study was 0.910 [26]. It also worth mentioned that the researchers in the present study take a permission to use the PCQ by Mind Garden (Order number = FHQGGJGOE, from/https://www.mindgarden.com).

### Ethical consideration

The approval of the Research Ethics Committee (REC), Faculty of Nursing, Alexandria University, was obtained for the study settings to gather the necessary data. The aim of the study was clarified to the participating nurses through emails and What’s App was assured that all data would be used for research purposes only and each one was informed of the right to refuse participation in the study or withdraw at any time before completing the study tools with no consequences. Informed written consent was obtained from nursing educators who accepted to participate in the study. Anonymity was considered and respected. Data confidentiality was assured during the implementation of the study**.**

### Data collection phase

Official permission from the faculty of nursing authorities at Alexandria University, Egypt, was obtained to conduct the study. A pilot study was done to assess the study instruments’ applicability, clarity, and practicality. It was carried out on 25 nursing educators who were not included in the study’s participants. The pilot’s findings revealed that no changes were needed. The internal consistency of the research instruments was assessed using the Alpha Cronbach’s test showed good reliability for the study tools. As well as the data was collected from November 2021 to February 2022.

### Statistical analysis

The collected data were summarized and presented in tables. Continuous non-normally distributed variables were presented as a median and Interquartile range (IQR), and categorical variables were presented as numbers and percentages. The Spearman correlation evaluated the relationship between the spiritual leadership and psychological capital scales. The correlation coefficient was interpreted as low correlation, moderate correlation, and strong correlation. *P*-values ≤0.05 were considered statistically significant. The multinomial logistic regression model was used for spiritual leadership and psychological capital scales, and the reference category was “low”. The previous tests were conducted using statistical analyses using the Statistical Package for the Social Sciences (SPSS) software, version 28.0 (IBM Corp., Armonk, NY, USA).

## Results

The results reflected 215 responses; two participants were excluded due to not being accepted to participate in our study. They left 213 responses included in our analysis.

Table 1 showed that the majority of the nursing educators are females (91.5%), and about two-thirds of study participants were married (70.4%). While more than three-fifths of them are married (70.4%). Moreover, more than half of the study-nursing educators are Ph.D. Graduates (54.9%).

**Table 1:** Distribution of the Studied Nurses Educators according to Demographic Data *(N = 213)*

Variables		***N***	***%***
**Age**	Median; IQR	34	21
**Gender**	Male	18	8.5
	Female	195	91.5
**Marital Status**	Single	50	23.5
	Married	150	70.5
	Divorced	1	0.5
	Widow	12	5.5
**Educational Level**	PhD	117	55
	Master’s Degree	63	29.5
	Higher Diploma after Baccalaureate	2	1
	Bachelor of Nursing	31	14.5
**Scientific Degree**	Professor	37	17.5
	Assistant Professor	36	16.75
	Lecturer	36	16.75
	Assistant Lecturer	35	16.5
	Demonstrator	35	16.5
	Practical guides	34	16
**Department**	Nursing Management	23	11
	Psychiatric Nursing and Mental Health	21	10
	Nursing Education	21	10
	Pediatric Nursing	17	8
	Critical Care and Emergency Nursing	26	12
	Maternity, Gynecology, and Obstetrics Nursing	24	11.25
	Internal and Surgical Nursing	42	19.25
	Elderly Nursing	16	7.5
	Community Health Nursing	23	11
**Number of Years of Experience Within the Department**	Less than five years	45	21
	10–5 years old	51	24
	10–15 years old	40	19
	More than 15 years	77	36
**Number of Years of Experience Since Graduation**	Less than five years	77	36
	10–5 years old	48	22.5
	10–15 years old	28	13.5
	More than 15 years	60	28

N: Number %: Percentage IQR: Interquartile Range PhD: Doctor of Philosophy

The overall SL level is moderate (49.8%). Regarding nursing educators’ subscales, the highest percentage was reported on the “altruistic love” subscale (54%), while the lowest one was reported on the “hope and faith” subscale (28.6%). The largest percentage of respondents (65.3%) who scored an individual subscale as “high level” was for the “hope and faith” subscale. Moreover, the lowest percentage was reported on the “altruistic love” subscale (25.8%). The largest percentage of respondents (20.2%) who scored an individual subscale as “low level” was for the “altruistic love” subscale. In addition, the lowest percentage was reported on the “hope and faith” subscale (6.1%) (Table 2).

**Table 2:** Spiritual Leadership Scale Dimensions Score and Grades *(N = 213)*

Spiritual leadership Scale Dimensions	Score		Grades					
			Low		Moderate		High	
	Median	IQR	N	%	N	%	N	%
**Vision**	18	5	26	12.2	94	44.1	93	43.7
**Hope and Faith**	20	4	13	6.1	61	28.6	139	65.3
**Altruistic Love**	21	9	43	20.2	115	54	55	25.8
**Overall Spiritual leadership Scale**	59	16	25	11.7	106	49.8	82	38.5

N: Number %: Percentage IQR: Interquartile Range

Table 3 shows that the overall psychological capital level is high (63.4%). Regarding the nursing educators subscales, the highest percentage was reported on the “self-efficacy” subscale (67.6%), while the lowest one was reported on the “optimism” subscale (46.9%). The largest percentage of respondents (5.2%) who scored an individual subscale as “low level” was for the “hope” subscale. Moreover, the lowest percentage was reported on the “resilience” subscale (3.3%). While the largest percentage of respondents (49.3%) who scored an individual subscale as “moderate level” was for the “optimism” subscale. And the lowest percentage was reported on the “self-efficacy” subscale (28.6%).

**Table 3:** Psychological Capital Scale Dimensions Score and Grades *(N = 213)*

Psychological Capital Scale Dimensions	Score		Grades					
			Low		Moderate		High	
	Median	IQR	N	%	N	%	N	%
**Self-Efficacy**	24	5	8	3.8	61	28.6	144	67.6
**Hope**	28	6	11	5.2	68	31.9	134	62.9
**Resilience**	20	4	7	3.3	65	30.5	141	66.2
**Optimism**	22	5	8	3.8	105	49.3	100	46.9
**Overall Psychological Capital Scale**	93	18	5	2.3	73	34.3	135	63.4

*N* Number, *%* Percentage, *IQR* Interquartile Range

Table 4 revealed a strong, positive, and significant correlation were noted between overall spiritual leadership scale and overall psychological capital scale (R-value = 0.635 and *p*-value = 0.001). Moderate, positive, and significant correlations were noted between optimism and vision (Rho(R)-value = 0.419 and *p*-value = 0.001), hope and faith (R-value = 0.441 and p-value = 0.001), altruistic love (R-value = 0.302 and p-value = 0.001), and overall spiritual leadership scale (R-value = 0.406 and p-value = 0.001). At the same time, strong, positive, and significant correlations were noted between hope and vision (R-value = 0.644 and p-value = 0.001), hope and faith (R-value = 0.746 and p-value = 0.001), altruistic love (R-value = 0.523 and p-value = 0.001), and overall spiritual leadership scale (R-value = 0.660 and p-value = 0.001). Strong, positive, and significant correlations were noted between self-efficacy and vision (R-value = 0.610 and p-value = 0.001), hope and faith (R-value = 0.678 and p-value = 0.001), altruistic love (R-value = 0.506 and p-value = 0.001), and overall spiritual leadership scale (R-value = 0.629 and p-value = 0.001).

**Table 4:** Correlation Matrix between Spiritual Leadership Scale and Psychological Capital Scale

Spiritual leadership Scale		Psychological Capital Scale				
		Self-Efficacy	Hope	Resilience	Optimism	Overall
**Vision**	Correlation Coefficient	0.610	0.644	0.537	0.419	0.622
	P-Value	0.001	0.001	0.001	0.001	0.001
**Hope and Faith**	Correlation Coefficient	0.678	0.746	0.655	0.441	0.715
	P-Value	0.001	0.001	0.001	0.001	0.001
**Altruistic Love**	Correlation Coefficient	0.506	0.523	0.421	0.302	0.497
	P-Value	0.001	0.001	0.001	0.001	0.001
**Overall**	Correlation Coefficient	0.629	0.660	0.552	0.406	0.635
	P-Value	0.001	0.001	0.001	0.001	0.001

Spearman Correlation *P*-values ≤0.05 were considered statistically significant

Table 5 pointed that having a Ph.D. Was expected to have high agreement on overall spiritual leadership questions 1.87 times (95% confident interval (CI) [0.428–8.207]) more than having a Bachelor of Nursing. Being a professor predicted having high and moderate agreement on overall spiritual leadership questions 11.903 and 2.764 times more than a practical guide, respectively. Being an assistant professor, lecturer, assistant lecturer, and demonstrator predicted having high agreement on overall spiritual leadership questions 1.450, 4.529, 3.479, and 3.941 times more than being a practical guide. From 10 to 15 years of experience within the department was expected to have high and moderate agreement on overall spiritual leadership questions 1.124 and 1.594 times more than having more than 15 years of experience within the department, respectively (Table 5). Scores of vision hope and faith, altruistic love, self-efficacy, and hope were significant predicators for high and moderate agreement on overall spiritual leadership questions (*p*-value < 0.05). Low and moderate self-efficacy, hope, and resilience were significant predicators for high and moderate agreement on overall spiritual leadership questions (p-value < 0.05). The score of the psychological capital scale was a significant predictor of high and moderate agreement on overall spiritual leadership questions (p-value < 0.05).

**Table 5:** Multinomial Logistic Regression Model between Socio-Demographic, Clinical Data, and Spiritual Leadership Scale

Variables		Moderate				High			
		P-value	Odds Ratio	95% Confidence Interval		P-value	Odds Ratio	95% Confidence Interval	
				Lower	Upper			Lower	Upper
**Age**		0.**214**	0.959	0.899	1.024	0.**709**	0.986	0.913	1.064
**Gender**	Male	0.**476**	0.521	0.087	3.134	0.**869**	0.858	0.**140**	5.271
	Female	**Ref**				**Ref**			
**Educational Level**	PhD	0.605	0.695	0.175	2.757	0.404	1.875	0.428	8.207
	Master’s Degree	0.347	0.510	0.125	2.075	0.665	0.712	0.154	3.297
	Higher Diploma after Baccalaureate	0.**219**	0.150	0.007	3.092	0.141	0.717	0.**461**	1.116
	Bachelor of Nursing	**Ref**				**Ref**			
**Scientific Degree**	Professor	**0.502**	2.764	0.142	53.721	**0.110**	11.903	0.572	247.695
	Assistant Professor	**0.467**	0.437	0.047	4.069	**0.754**	1.450	0.142	14.819
	Lecturer	**0.315**	2.422	0.432	13.584	**0.089**	4.529	0.795	25.811
	Assistant Lecturer	**0.033**	5.782	1.152	29.019	**0.136**	3.479	0.676	17.921
	Demonstrator	**0.010**	14.189	1.884	106.865	**0.210**	3.941	0.463	33.577
	Practical Guide	**Ref**				**Ref**			
**Number of Years of Experience Within the Department**	Less than 5 years	**0.345**	0.248	0.014	4.473	**0.905**	0.835	0.043	16.344
	10–5 years old	**0.278**	0.199	0.011	3.670	**0.705**	0.566	0.030	10.795
	10–15 years old	**0.737**	1.594	0.105	24.102	**0.934**	1.124	0.070	17.989
	More than 15 years	**Ref**				**Ref**			
**Number of Years of Experience Since Graduation**	Less than 5 years	**0.743**	0.733	0.115	4.669	**0.481**	0.512	0.079	3.297
	10–5 years old	**0.685**	0.607	0.055	6.742	**0.774**	0.699	0.061	8.024
	10–15 years old	**0.360**	0.299	0.022	3.966	**0.614**	0.510	0.037	6.965
	More than 15 years	**Ref**				**Ref**			
**Vision**	Score	**0.001**	2.767	1.788	4.283	**0.001**	12.068	6.381	22.825
**Hope and Faith**	Score	**0.008**	2.464	1.265	4.801	**0.001**	18.457	5.347	63.714
**Altruistic Love**	Score	**0.001**	2.402	1.399	4.125	**0.001**	9.631	4.200	22.087
**Self-Efficacy**	Low	**0.254**	0.148	0.006	3.945	**0.001**	9.762	9.762	9.762
	Moderate	**0.491**	0.588	0.130	2.657	**0.001**	0.041	0.011	0.153
	High	**Ref**				**Ref**			
	Score	**0.052**	1.234	0.998	1.525	**0.005**	1.445	1.118	1.868
**Hope**	Low	**0.019**	0.044	0.003	0.597	**0.001**	5.609	5.609	5.609
	Moderate	**0.094**	0.228	0.040	1.287	**0.001**	0.039	0.011	0.143
	High	**Ref**				**Ref**			
	Score	**0.143**	1.145	0.955	1.371	**0.001**	1.521	1.206	1.920
**Resilience**	Low	**0.812**	1.739	0.018	164.914	**0.001**	0.021	0.002	0.201
	Moderate	**0.389**	1.904	0.440	8.246	**0.001**	0.035	0.009	0.133
	High	**Ref**				**Ref**			
	Score	**0.313**	0.872	0.668	1.138	**0.166**	0.801	0.585	1.097
**Optimism**	Low	**0.291**	0.195	0.009	4.073	**0.001**	1.503	1.503	1.503
	Moderate	**0.492**	1.672	0.387	7.226	**0.092**	0.407	0.143	1.160
	High	**Ref**				**Ref**			
	Score	**0.963**	1.004	0.847	1.190	**0.823**	0.977	0.798	1.196
**Psychological Capital Scale**	Score	**0.001**	1.069	1.034	1.106	**0.001**	1.179	1.125	1.236

PhD: Doctor of Philosophy Ref: Reference P-values ≤0.05 were considered statistically significant. The reference category is (Low Grade

Table 6 showed that being a male was expected to have high and moderate agreement on overall psychological capital questions 1.563 and 1.600 times more than being a female. Being single was predicted to have high and moderate agreement on overall psychological capital questions 2.299 and 3.924 times more than being a widow. Being divorced was predicted to have high and moderate agreement on overall psychological capital questions 2.373 and 76.471 times more than a widow. Having a Ph.D. was expected to have high agreement on overall psychological capital questions 3.387 times (95% CI [0.043–263.865]) more than having a Bachelor of Nursing. The participants with a higher Diploma after Baccalaureate were expected to have moderate agreement on overall psychological capital questions 1.260 times (95% CI [0.570–2.786]) more than those having a Bachelor of Nursing. Belonged to the pediatric nursing department was predicted to have high and moderate agreement on overall psychological capital questions 5.071 and 5.757 times more than community health nursing. Scores of vision hope and faith, and spiritual leadership scale were significant predictors for high agreement on overall psychological capital questions (*p*-value < 0.05). Having a master’s degree, being an assistant lecturer, being a demonstrator, having less than 5 years of experience within the department, and all items of having experience since graduation were significant predicators for high and moderate agreement on overall psychological capital questions (p-value < 0.05).

**Table 6:** Multinomial Logistic Regression Model between Socio-Demographic, Clinical Data, and Psychological Capital Scale

Variables		Moderate				High			
		P-value	Odds Ratio	95% Confidence Interval		P-value	Odds Ratio	95% Confidence Interval	
				Lower	Upper			Lower	Upper
**Age**		0.269	1.137	0.905	1.428	0.086	1.221	0.972	1.533
**Gender**	Male	0.800	1.600	0.042	60.527	0.808	1.563	0.043	57.210
	Female	**Ref**				**Ref**			
**Marital Status**	Single	0.622	3.924	0.017	893.331	0.756	2.299	0.012	435.948
	Married	0.813	0.552	0.004	74.543	0.695	0.390	0.003	43.590
	Divorced	0.810	76.471	3.406	17.1708	0.962	2.373	6.132	91.809
	Widow	**Ref**				**Ref**			
**Educational Level**	PhD	0.738	0.462	0.005	42.444	0.583	3.387	0.043	263.865
	Master’s Degree	0.008	0.005	0.000	0.251	0.020	0.010	0.000	0.484
	Higher Diploma after Baccalaureate	0.568	1.260	0.570	2.786	0.120	0.526	0.234	1.183
	Bachelor of Nursing	**Ref**				**Ref**			
**Scientific Degree**	Professor	0.466	0.084	0.000	64.483	0.428	0.073	0.000	47.518
	Assistant Professor	0.463	0.096	0.000	49.916	0.430	0.086	0.000	38.156
	Lecturer	0.149	0.040	0.001	3.146	0.070	0.022	0.000	1.359
	Assistant Lecturer	0.000	1039.872	22.087	48,957.034	0.001	536.191	12.850	22,373.310
	Demonstrator	0.000	9726.743	115.081	822,109.603	0.000	2950.992	37.246	233,804.920
	Practical Guide	**Ref**				**Ref**			
**Department**	Nursing Management	0.861	1.345	0.048	37.522	0.600	2.453	0.086	69.990
	Psychiatric Nursing and Mental Health	0.975	0.940	0.021	41.464	0.807	1.608	0.036	72.614
	Nursing Education	0.914	0.820	0.023	29.783	0.508	3.262	0.098	108.420
	Pediatric Nursing	0.318	5.757	0.185	178.796	0.357	5.071	0.160	161.022
	Critical Care and Emergency Nursing	0.847	0.715	0.024	21.711	0.824	1.467	0.050	43.100
	Maternity, Gynecology, and Obstetrics Nursing	0.539	0.367	0.015	8.993	0.847	1.362	0.059	31.547
	Internal and Surgical Nursing	0.135	0.084	0.003	2.153	0.089	0.059	0.002	1.538
	Elderly Nursing	0.816	0.621	0.011	34.094	0.775	1.779	0.034	92.107
	Community Health Nursing	**Ref**				**Ref**			
**Number of Years of Experience Within The Department**	Less than 5 years	0.010	0.000	1.045	0.113	0.012	0.000	2.271	0.157
	10–5 years old	0.233	0.019	2.971	12.600	0.238	0.023	4.443	12.020
	10–15 years old	0.682	3.476	0.009	1334.387	0.658	3.688	0.011	1187.497
	More than 15 years	**Ref**				**Ref**			
**Number of Years of Experience Since Graduation**	Less than 5 years	0.000	9.637	7.447	1.247	0.000	2.088	1.771	2.461
	10–5 years old	0.000	4.551	3.436	6.027	0.000	1.553	1.308	1.843
	10–15 years old	0.000	5.354	1.902	1.507	0.000	1.820	1.820	1.820
	More than 15 years	**Ref**				**Ref**			
**Vision**	Score	0.005	1.867	1.206	2.889	0.000	2.622	1.668	4.120
**Hope and Faith**	Score	0.210	1.836	0.710	4.747	0.020	3.262	1.205	8.832
**Altruistic Love**	Score	0.281	1.184	0.871	1.609	0.090	1.311	0.959	1.793
**Self-Efficacy**	Score	0.660	1.274	0.433	3.746	0.415	1.563	0.534	4.580
**Hope**	Score	0.468	1.466	0.522	4.122	0.356	1.623	0.581	4.539
**Resilience**	Score	0.448	1.580	0.485	5.141	0.245	2.015	0.618	6.569
**Optimism**	Score	0.643	1.246	0.492	3.155	0.235	1.759	0.692	4.473
**Spiritual Leadership Scale**	Score	0.052	1.266	0.998	1.607	0.003	1.448	1.132	1.853

PhD: Doctor of Philosophy Ref: Reference P-values ≤0.05 were considered statistically significant. The reference category is (Low Grade)

## Discussion

Recently, leadership studies changed direction, and they have started to focus on ethical, spiritual, and more human-focused versions of leadership aiming at justice, participation, and glorifying employees, rather than hierarchical chief-officer relationships [27]. Overall, nursing educators perceived their leadership behaviors as moderate level. This result is in line with a recent **studies** done by Abouzaid, (2019) and Ali, Ibrahim, & Diab, (2021) who reported that the total levels of spiritual leadership among nurse leaders were between moderate to high [28, 29]. As well as Ali, Ibrahim, & Diab, (2021**)** showed that nursing managers’ spiritual leadership levels were high on the level of meaning/calling, vision, and hope/faith dimensions while the largest percentage of participants who scored an individual subscale as “low level” was for the “altruistic love” subscale [29].

The total score for psychological capital in the present study was high. This may have occurred because there was an effective organizational climate, effective communication between nursing educators and their managers, or a low level of perceived occupational stress. This result was supported by the studies of Çelik, (2018) and Ibrahim, Elwekel, Osman, & El-Gilany, (2020), who found that the majority of the nurses exhibited high levels of psychological capital [30, 31]. This result disagreed with a study done by Metwaly & Ahmed, (2018), who revealed that the studied nurses had a low level of psychological capital. The current results also showed that the highest percentage was reported on the “self-efficacy” subscale, while the lowest one was reported on the “optimism” subscale [32]. In the same line, Percunda & Putri, (2020) found that the nurse participants have high scores in self-efficacy and hope, followed by optimism and resilience [33].

The present study revealed a significant positive correlation between spiritual leadership practices and psychological capital; with another meaning, the researchers in the present study found that the more nursing educators reported spiritual leadership, the more they reported psychological capital. This may be explained by the fact that when nurse educators have a clear sense of purpose in their profession, they feel joy, contentment, and self-worth at work. *Fredrickson’s* broaden-and-build theory of positive emotions serves as the theoretical foundation for the hypothesized relationship between spiritual leadership and psychological capital. Fredrickson claims that nurturing happy feelings at work leads to happiness [34].

Relevant studies have revealed that positive psychological capital and appropriate leadership styles, especially spiritual leadership, can revolutionize the nursing environment, promote retention, increase productivity, enhance their positive psychological state, and provide meaning and purpose in work for nurses and nurse educators [35, 36]. These findings highlight the need to develop strategies for improving spiritual leadership among nursing educators through education, training, and policy development.

Other studies have shown a positive correlation between spiritual leadership practices and psychological capital; Chen and Li, (2013) reported that spiritual leadership is positively related to employees’ self-esteem and self-efficacy, which were considered two of the main components of psychological capital [15]. In another study done by Wu, & Lee, (2020) about the effect of spiritual leadership on nurses’ work engagement and psychological capital, they found that the more the studied nurses reported high spiritual leadership, the more they experienced high psychological capital [22].

The new phenomenon of spiritual leadership in organization management that focuses on the humanistic values of the organization’s followers, like; intimacy, honesty, love, emotion, generosity, cooperation, hope, kindness, and altruism, can be developed in managerial activities. All of these activities will develop a sense of belonging and self-identity among the organization’s employees, which will be reflected in their self-efficacy. The results also showed that spiritual leadership positively correlates with nursing educators’ self-efficacy. That was consistent with a study done by Faghih Aram, (2017) in Iran between high school managers and staff; she found that spiritual leadership had a significant positive relationship with the self-efficacy of managers and employees. In addition, the overlook of the love of altruism, faith in work, organizational membership, organizational commitment, and significant improvements in work productivity showed a significant relationship with efficacy [37].

In addition, the positive correlation between spiritual leadership, hope, and optimism of psychological capital can be justified as nurses who work under spiritual leaders are more likely to achieve work goals, and successful work experiences lead to increased motivation and optimism in their jobs. The most unexpected finding in the current study was that male participants were expected to have more spiritual leadership and psychological capital than females, even with the small number of male educators who participated in the present study. This may be related to the fact that male educators have greater wisdom than female educators and have a perceptive and wise perspective [38].

Moreover, Radu, (2017) reported that males have characteristics that make them better at leadership. These criteria include tradition (accumulating information based on prior experience), innovation (being open to new ideas and willing to take risks), strategy (seeing the big picture), calmness (tending to keep their emotions in check), delegation (assigning goals and responsibilities), cooperation (being good teammates), and persuasion (they sell ideas and win people over) [39]. In addition, nursing educators who have a Ph.D. Education was expected to have higher agreement on spiritual leadership and psychological capital than those with a bachelor’s degree in nursing. Nursing educators who were assistant lecturers and demonstrators are expected to have more spiritual leadership and psychological capital than other degrees.

## Limitations

This research has several limitations. First, causality cannot be determined because this study used a cross-sectional methodology. As a result, a subsequent study using a more rigorous research technique may be considered. Due to the use of self-reported measures, response bias may exist. Second, the study’s sample size was limited, which limited the results’ application and weakened the statistical power. To identify causal links and differences across variables, longitudinal empirical investigations with large samples are necessary. More study is needed to see how these spiritual and psychological capital practices affect nursing educators’ health outcomes and treatment quality.

## Conclusion

In general, the findings of this study showed that nursing educators had moderate to high levels of spiritual leadership and psychological capital. In addition, there is a strong, positive, and highly significant correlation between spiritual leadership and psychological capital. Finally, although some socio-demographic and clinical data of nursing educators reflected an expected agreement with spiritual leadership and psychological capital, these factors need to be assessed in the future with more focus and analysis.

### Implications for nursing management

Our study results suggest that spiritual leadership is pivotal in promoting followers’ psychological capital. Therefore, our work validates the importance of developing and practicing spiritual leadership to foster followers’ psychological capital. Nursing leaders must have spiritual competencies and must include the promotion of workplace spirituality on the daily agenda as a foundational area in management. As well as the healthcare institutions’ managers, they should also consider the best leaders who facilitate workplace spirituality.

## Supplementary Information

**Additional file 1.** Survey

## Data Availability

The datasets used and/or analyzed during the current study available from the corresponding author on reasonable request.
