# Supplementary material for: Spiritual leadership among nursing educators: a correlational cross-sectional study with psychological capital
Source: BMC Nurs. 2022 Dec 31;21:377. doi: 10.1186/s12912-022-01163-y (PMC9805246; doi:10.1186/s12912-022-01163-y)
Supplement: Supplementary file 1 — Additional file 1. Survey [file 12912_2022_1163_MOESM1_ESM.docx]

Spiritual Leadership among Nursing Educators: A Correlational Cross-Sectional Study with Psychological Capital

1- Do you accept to participate in the survey?

Yes ( ) No ( )

Socio-demographic characteristics:

2- Gender Male ( ) female ( )

3- Age in years ……………….

4- Marital status

Married ( ) single ( ) divorced ( ) widowed ( )

5- Qualifications

PHD in nursing ( ) Master degree in nursing ( ) Bachelor degree in nursing ( )
High Nursing Institute ( ) Nursing Diploma ( )

6- Academic position

- Clinical Instructor
- Demonstrator
- Assistant Lecturer

- Lecturer
- Assistant Professor
- Professor

7. Department (Specialty)

- Medical and Surgical Nursing
- Pediatric Nursing
- Maternity, Obstetrics and Gynecology Nursing
- Psychiatric and Mental Health Nursing
- Nursing Administration
- Family and Community Health Nursing
- Nursing education
- Geriatric nursing
- Critical care and emergency nursing

8. Years of experiences in current position

( ) < 5 years ( ) 5-10 years ( ) 11-20 years ( ) more than 20 years

9. Years of experiences since bachelor of nursing graduation

( ) < 5 years ( ) 5-10 years ( ) 11-20 years ( ) more than 20 years

I. Spiritual leadership Scale

<u>Spiritual leadership Scale</u>	Full agreement 5	agree 4	neutral 3	disagree 2	Full disagreement 1
**A. Vision: describes the organization's journey and why we are taking it; defines who we are and what we do.**					
1. I understand and am committed to my organization's vision.					
2. My workgroup has a vision statement that brings out the best in me.					
3. My organization's vision inspires my best performance					
4. I have faith in my organization's vision for its employees					
5. My organization's vision is clear and compelling to me.					
**B. Hope/faith: the assurance of things hoped for, the conviction that the organization's vision/purpose/mission will be fulfilled.**					

1. I have faith in my organization and I am willing to do whatever it takes to insure that it accomplishes its mission.					
2. I persevere and exert extra effort to help my organization succeed because I have faith in what it stands for.					
3. I always do my best in my work because I have faith in my organization and its leaders.					
4. I set challenging goals for my work because I have faith in my organization and want us to succeed					
5. I demonstrate my faith in my organization and its mission by doing everything I can to help us succeed					
**C. Altruistic love: a sense of wholeness, harmony, and well-being produced through care, concern, and appreciation for both self and others**					
1. My organization really cares about its people.					
2. My organization is kind and considerate toward its workers, and when they are suffering, wants to do					

something about it.					
3 .The leaders in my organization walk and walk as well as talk and talk_					
4. My organization is trustworthy and loyal to its employees.					
5. My organization does not punish honest mistakes.					
6. The leaders in my organization are honest and without false pride.					
7. The leaders in my organization have the courage to stand up for their people					

II. Psychological Capital Scale

**Psychological Capital Scale**	**Very ineffective 1**	**Ineffective 2**	**Neither effective nor ineffective 3**	**Effective 4**	**Very effective 5**
**A. Self-efficacy**					
1.I feel confident when I´m looking for a solution to a long-term problem					
2 .I feel confident in representing my work area in meetings with the organization management					
3. I feel confident to contribute to discussions about the organization´s strategy					
4. I am able to define set goals for my work area					
5. I feel confident when I need to make contact with people outside the company (e.g. customers and suppliers) to discuss					

problems					
6. I feel confident to present information to a group of colleagues					
**B. Hope**<br>7. If I were in a difficult situation at work, I could think of many ways to get out of it					
8. Nowadays, I try to achieve my goals with great energy					
9. For any problem, there are many ways to solve it					
10. Right now, I see myself as a successful person at work					
11. I can think of many ways to achieve my goals at work					
12. Right now I am achieving the professional goals that I defined for myself					
13. When I have a setback in class/in the					

flight simulator/flying, I have trouble recovering from it, moving on					
**C. Resilience** 14. In one way or another, in general I can manage work and its difficulties					
15. At work, if necessary, I am able to stand "at my own risk"					
16. In general, I can easily step over the more stressful things at work					
17. I can overcome the difficult times at work, because I already came through difficulties in the past					
18. I feel that I can handle many things at the same time at work					
**D. Optimism** 19. When things are uncertain for me at work, I usually expect the best					

20. If something can go wrong for me training-wise, it will					
21. In my work, I always look on the positive side of things					
22. At work, I am optimistic about what will happen in the future					
23. At work, things never go as I would like					
24. I work with the conviction that every setback has a positive side					
